# Association between socio-ecological factors and leisure time physical activity (LTPA) among older adults in Sichuan, China: a structural equation modeling analysis

**DOI:** 10.1186/s12877-021-02730-9

**Published:** 2022-01-18

**Authors:** Yufei Wang, Nanyan Li, Jingjie Zhu, Qian Deng, Julinling Hu, Jue Xu, Junmin Zhou

**Affiliations:** West China School of Public Health and West China Fourth Hospital, Sichuan University, Chengdu, 610041 Sichuan China

**Keywords:** Individual correlates, Interpersonal correlates, Social correlates, Environmental correlates, Aging, Leisure time physical activity

## Abstract

**Background:**

Few studies examined socio-ecological factors and leisure time physical activities (LTPA) and rarely focused on self-regulation and social capital, which might play a significant role in impacting people’s physical activity behavior. This study aimed to examine the direct and indirect effects of individual level (perceived benefits, perceived barriers, and self-efficacy), interpersonal level (self-regulation), social level (social capital), and environmental level factors (perceived physical environment) on LTPA among older adults.

**Methods:**

A cross-sectional study was conducted in 737 older adults from Sichuan, China. Structural equation modeling (SEM) analysis was used to examine the associations of individual, interpersonal, social, and environmental level factors with LTPA.

**Results:**

The mean age of all participants was 71.22 (range, 60–97), and 56.1% of them were women. The SEM results showed that individual level variables (β = 0.32, ρ < 0.001), self-regulation (β = 0.18, ρ < 0.001) and social capital (β = 0.14, ρ < 0.001) could all directly affect LTPA while there was no significant association of perceived physical environment with LTPA. Self-regulation served as a bridge linking social capital and LTPA. Individual level variables contributed the largest total effect (0.32) on LTPA. Self-regulation and social capital had the same total effect (0.18) on LTPA.

**Conclusions:**

Factors on three levels were all significantly associated with LTPA. Interventions that incorporate individual, interpersonal, social factors may be considered to promote LTPA in older adults. Self-regulation should receive more attention in future interventions.

**Supplementary Information:**

The online version contains supplementary material available at 10.1186/s12877-021-02730-9.

## Background

Severe population aging is projected to occur in most parts of the world in the next several decades [1]. In China, the proportion of the population aged 60 years or older reached 13.3% in 2010 and it is projected to increase to 19.3% by 2025 [2]. Such an increase will undoubtedly result in a huge burden upon healthcare systems. As one of the most important modifiable lifestyles, physical activity plays a vital role in health promotion strategies aimed to reduce healthcare expenditures while improving the quality of later life [3]. Thus, a better understanding of the factors that influence physical activity in older adults appears to be necessary.

The socio-ecological model suggests that behaviors are influenced by an interaction of individual, interpersonal, social, and environmental level factors [4]. A few studies have investigated the correlates of physical activity using the socio-ecological model [5–7]. Specifically, individual level factors, such as self-efficacy, perceived benefits, perceived barriers, have been demonstrated to be important factors influencing physical activity [6, 7]; social norm, as an interpersonal level factor, is found to be associated with physical activity [8]; social support, either at interpersonal level or at social level, is considered to be one of the determinants of physical activity [6, 8, 9]; environment has been suggested to be associated with physical activity [7, 9, 10]. However, few studies have examined the effects of self-regulation and social capital on physical activity in the socio-ecological framework. Self-regulation, as one of the interpersonal factors [11, 12], is associated with physical activity [13, 14], and may mediate the relationship between social factors and physical activity [14]. Social capital, defined as a set of social contacts that enable access to social, emotional, and practical support [15], is also associated with physical activity [16] and impacts physical activity indirectly by influencing other variables [17]. Given self-regulation and social capital’s significant effects on physical activity and their potential interaction with other variables, it appears to be necessary to examine the self-regulation, social capital, and other correlates of physical activity using the socio-ecological model.

Furthermore, despite that correlates of physical activity have been widely examined using the socio-ecological model, little is known about the socio-ecological factors of leisure time physical activity (LTPA), the most important domain of physical activity [18], especially in older adults [19]. Thus, a study focusing on socio-ecological correlates of LTPA among older adults is warranted. The aim of our study was to examine whether individual level (perceived benefits and barriers, self-efficacy), interpersonal level (self-regulation), social level (social capital), and environmental level (perceived physical environment) factors were associated with LTPA among older adults in China, considering both direct and indirect effects.

## Methods

### Participants and data collection

Data used in the study comes from a cross-sectional design, population-based survey conducted in October 2020. A multi-stage random sampling method was used to recruit a sample aged 60 years and older in Sichuan Province, western China. First, Chengdu was randomly selected from 18 cities of Sichuan Province. Second, Jianyang was randomly selected among 20 regions in Chengdu. Jianyang is located in the western part of the Sichuan Basin (area: 2213.5km^2^, villiage:853, population:1,171,200). Third, four villages (Jianzheng, Guilin, Yixue, and Qianfeng) were randomly selected in the rural areas of the city. Random selections were generated by a computer program (www.random.org). Fourth, a total of 737 participants (aged from 60 to 97 years) were randomly recruited from the four villages. Twenty-four of them were excluded due to immobile/deaf, severe mental disease, or incomplete identifying information. All of the eligible participants were invited to a face-to-face survey, which usually took 20–30 min. Informed consent was obtained from each participant before conducting the survey. The study protocol was approved by the Sichuan University Medical Ethical Review Board (K2019073).

### Instruments

#### Leisure time physical activity (LTPA)

The participant’s level of leisure time physical activity in the previous week was obtained by an Interview-based questionnaire, which is a part of the Physical Activity Scale for the Elderly (PASE) [20]. The score of LTPA is calculated by multiplying the weekly time (hours) spent in every single leisure time physical activity (mainly walking, light-intensity sport, moderate-intensity sport, strenuous-intensity sport, muscle strength exercise) with the activity weight. The weights had been obtained in previous validation studies [20, 21]. The reliability and validity have been supported in the older Chinese population [22] and the internal consistency of this study was acceptable (α = 0.646).

#### Sociodemographic variables

The survey included questions on sociodemographic factors including age, sex, marital status, education, employment status, and annual household income (RMB).

#### Individual level variables

Self-efficacy was assessed using a 9-item scale [23], rated from 0 (not confident) to 10 (very confident). Participants were asked to answer the questions in 9 different conditions, for example, “How confident are you that you could exercise three times per week and 20 minutes per time if the weather is not good?” The self-efficacy score was obtained by summing all items. The reliability and validity of the scale have been acceptable in a previous study [23] and the internal consistency of this study was 0.863.

Scales measuring perceived benefits (12 items) and barriers (6 items) were adapted from a previously validated scale, Exercise Benefits/Barriers Scale (EBBS) [24]. The example of items in the perceived benefits scale was “Exercise decreases feelings of stress and tension for me.” The example of items in the perceived barriers scale was “Exercising takes too much of my time.” A 5-point Likert scale measured the level of agreement with the given items (1 = strongly disagree, 5 = strongly agree). The Internal consistency of benefits and barriers scale in this study were 0.846 and 0.660, respectively.

#### Interpersonal level variable

Self-regulation was assessed with a scale adapted and validated for older adults [25]. The scale (12 items) including the following dimensions: self-monitoring, goal setting, eliciting social supporting, reinforcements, time management, and relapse prevention, using a 5-point response scale (“never = 1”, “rarely = 2”, “sometimes = 3”, “often = 4”, “very often = 5”). These items exhibited moderate inter-item correlation (Cronbach’s α = 0.792).

#### Social level variable

The instruments to assess social capital included the individual- and family- (IF-) based social capital scale and the community- and society- (CS-) based social capital scale [26]. The scales both used a 5-point Likert scale, rated from 1 (strongly disagree) to 5 (strongly agree). The IF-based social capital scale included seven items related to participants’ relationships and networks with family members, relatives, and friends. The CS-based social capital scale consisted of seven items, including participation in community activities, level of trust in health and community organizations, and so on. Both the IF-based social capital scale and the CS-based social capital scale were validated in the Chinese population [26, 27]. The internal consistency of the scales in our study was acceptable (α = 0.730).

#### Environmental level variable

Perceived physical environment consisted of seven separate items (1 = strongly disagree, 5 = strongly agree). Examples of the items were “You often see people out on walks in your neighborhood.” and “The streets are well lit.” The items used were adapted from a previous study [28]. The internal consistency of this study was 0.620, which was considered as acceptable.

### Statistical analyses

**Fig. 1:**
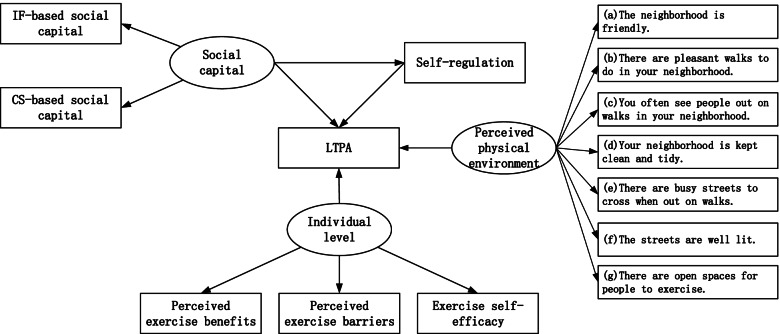
Socio-ecological model of LTPA

Mean and standard deviation were calculated for continuous variables. Frequency and percentage were calculated for categorical variables. Structural equation modeling (SEM) analysis was used to explore the associations of individual level, interpersonal level, social level, and environmental level factors with LTPA. The structural equation model included the path from individual level (latent variable, i.e., variables that are not directly observable) to LTPA; the path from self-regulation to LTPA; the path from social capital (latent variable) to LTPA; the path from perceived physical environment (latent variable) to LTPA; and the path from social capital (latent variable) to LTPA via self-regulation (Fig. 1). The comparative fit index (CFI), the goodness of fit index (GFI), the adjusted goodness of fit index (AGFI), and the root mean square error of approximation (RSMEA) were used to evaluate the model fit. The CFI (range, 0–1) measures how well the model fits, with higher values indicating better model fit [29]. The GFI and AGFI had to be > 0.95, the RMSEA had to be < 0.06 to accept a good fit [30]. Descriptive analyses and structural equation modeling analyses were performed with IBM SPSS Statistics version 23 and IBM Amos version 21, respectively.

## Results

Table 1 summarizes descriptive statistics for all variables. For sociodemographic characteristics, more than half of the participants were women (56.1%). The mean age of the sample was 71.22 (range, 60–97). The prevalence of employment was 62.4%. Most of the participants cohabited (72.7%). For individual level variables, participants’ perceived benefits scores (48.49 ± 6.85) were at a high level (range, 0–60). Individuals’ perceived barriers (12.60 ± 4.35, range 0–30) and self-efficacy (43.19 ± 25.23, range 0–90) scores were both at moderate levels. For the interpersonal level variable, the mean score of self-regulation (32.16 ± 10.29) was more than half of the total score, which ranges from 0 to 60. For the social level variable, the mean score of IF-based and CS-based social capital was 28.30 ± 4.47 and 24.17 ± 2.61, respectively. For environmental level variables, most items of perceived physical environment attained high scores (Mean > 3.5, range 0–5), except for “There are busy streets to cross when out on walks.” (2.73 ± 1.45).

**Table 1:** Sample studied variables (*N* = 731)

Variable	Mean ± SD or frequency (percentage)	Variable	Mean ± SD or frequency (percentage)
**Dependent variable**		**Individual level variables**	
Leisure time physical activity (LTPA)	31.94 ± 29.20	Perceived benefits	48.49 ± 6.85
**Sociodemographic variables**		Perceived barriers	12.60 ± 4.35
Gender		Self-efficacy	43.19 ± 25.23
Men	313 (43.9)	**Interpersonal level variable**	
Women	400 (56.1)	Self-regulation	32.16 ± 10.29
Age	71.22 ± 6.05	**Social level variable**	
Marital status		Social capital	
Cohabited	518 (72.7)	IF-based social capital	28.30 ± 4.47
Did not cohabit	195 (27.3)	CS-based social capital	24.17 ± 2.61
Education		**Environmental level variable**	
Illiteracy	473 (66.3)	Perceived physical environment	
Primary school	154 (21.6)	(a) The neighborhood is friendly.	4.32 ± 0.78
Middle school or above	86 (12.1)	(b) There are pleasant walks to do in your neighborhood.	4.31 ± 0.85
Employment		(c) You often see people out on walks in your neighborhood.	3.98 ± 1.16
Yes	445 (62.4)	(d) Your neighborhood is kept clean and tidy.	4.29 ± 0.73
No	268 (37.6)	(e) There are busy streets to cross when out on walks.	2.73 ± 1.45
Income (RMB)		(f) The streets are well lit.	3.62 ± 1.39
< 12,000	397 (55.7)	(g) There are open spaces for people to exercise.	3.51 ± 1.31
12,000–19,999	169 (23.7)		
≥20,000	147 (20.6)		

**Fig. 2:**
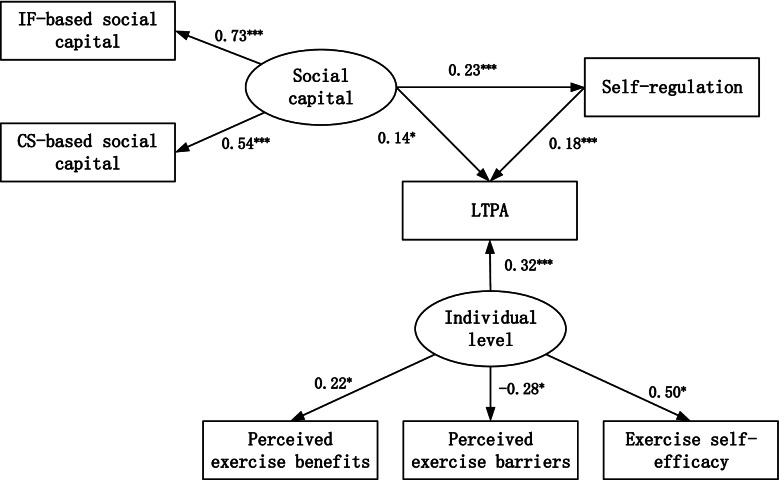
Effects of individual level variables, self-regulation and social capital on LTPA. Note: Only statistically significant paths are shown in the figure (Perceived physical environment was not included because it was not statistically significantly associated with LTPA.); *ρ < 0.05, ***ρ < 0.001

The structural equation model for LTPA in Fig. 2 demonstrated a good model fit (χ2/df = 2.617, GFI = 0.992, CFI = 0.977, AGFI = 0.971, RMSEA = 0.048). The two latent variables could be well-represented by their indicators. Specifically, for individual level variables, perceived benefits (β = 0.22, ρ < 0.05), perceived barriers (β = − 0.28, ρ < 0.05), and self-efficacy (β = 0.50, ρ < 0.05) were adequate indicators; social capital could be well-represented by the IF-based social capital (β = 0.73, ρ < 0.001) and CS-based social capital (β = 0.54, ρ < 0.001).

Table 2 showed the total effects of all variables on LTPA. The total effect of a given set of variables is the sum of its direct and indirect effects. For direct effects, individual level variables (β = 0.32, ρ < 0.001), self-regulation (β = 0.18, ρ < 0.001) and social capital (β = 0.14, ρ < 0.001) could all affect LTPA scores. The indirect effect of social capital on LTPA via self-regulation was 0.04 (0.23 × 0.18). Individual level variables contributed the largest total effect on LTPA with the standardized regression coefficient being 0.32. Self-regulation and social capital have the same total effect (0.18) on LTPA. Notably, perceived physical environment was not included in the model because it was not statistically significantly associated with LTPA (ρ > 0.05).

**Table 2:** Effects of individual level, self-regulation and social capital on LTPA

Variable	Direct Effect	Indirect Effect	Total Effect
Individual level	0.32	0.00	0.32
Self-regulation	0.18	0.00	0.18
Social capital	0.14	0.04	0.18

## Discussion

The study aimed to explore the factors influencing LTPA in older adults based on the framework of the socio-ecological model. The SEM analysis showed that individual level variables (self-efficacy, perceived benefits and barriers), interpersonal level variable (self-regulation), and social level variable (social capital) were significantly associated with LTPA, while environmental level variable (perceived physical environment) was not significantly associated with LTPA in older adults. Self-regulation mediated the pathway from social capital to LTPA. These socio-ecological factors may play an essential role when designing programs to promote leisure time physical activity for older adults.

Environmental factors were considered to be a key direction for future research on geriatric health [31]. Most studies have shown that some physical environmental factors (varies across studies) are associated with physical activity [32], especially in domains of accessibility of facilities [33, 34], aesthetics items [35], and so on. However, a handful of studies suggested that few environmental factors are associated with physical activity [7, 36], especially in LTPA [37]. Our results were consistent with the latter (An additional figure showed this in more detail (see Additional file 1)). The lack of significant correlation in our study could be partly due to two reasons. One reason could be that we integrated physical environment variables into one latent variable. This may obscure possible associations between individual physical environmental factors and physical activity [32]. The other one could be that there may be important environmental factors associated with physical activity in Chinese older adults that were not assessed in our scale (e.g., home equipment [36] and availability of walking trails [7]).

Existing literature suggested that social capital interventions were effective in improving social support [38], and high levels of perceived social support were associated with higher activity levels [39]. This may imply that social capital could be associated with physical activity, which has been proved in adolescents [16, 40]. However, we didn’t know if such associations exist among older adults. Our results that stronger social capital was associated with higher LTPA in older participants corroborated such speculation.

Self-regulation, as one of the most important factors to translate physical activity intention into action [13], was found in this study to be not only a direct factor affecting LTPA but also a mediating factor linking social capital and LTPA (self-regulation was not a significant mediator linking individual level variables and LTPA, see Additional file 2 for more details). Few studies have examined possible mechanisms explaining the link between social capital and physical activity, especially in the older population [17]. One of the potential mechanisms underlying the path from social capital to LTPA via self-regulation could be like this: Social capital, a set of social connections that give access to social, emotional, and practical support, makes people easier to obtain help from the social network [15]. Thus people with stronger social capital might be more likely to have access to professional exercise and time management methods to set goals and monitor themselves [41], which further improve their LTPA.

The study indicated that individual level variables had the largest total effect on LTPA, with self-efficacy having the greatest impact on individual level. In other words, self-efficacy was one of the most influential factors affecting LTPA in our study, and it has been widely acknowledged by previous literature [6, 7, 19, 42]. In addition, the finding of this study demonstrated perceived benefits and barriers have positive and negative effects on LTPA, respectively. This is in line with previous studies on physical activity [43, 44]. These findings highlight the importance of individual level variables on LTPA.

Several limitations of the study are noteworthy. First, our data were based on self-report from older adults, recall bias may not be avoided. Second, the cross-sectional design does not allow us to infer causality, warranting studies with longitudinal designs to verify such findings. Third, physical activities other than LTPA (i.e., occupational physical activity, transport activity, and housework) were not controlled for, which may overestimate the associations. Fourth, both intrapersonal and interpersonal dimensions are important in self-regulation [45], but this study was focused only on interpersonal dimension of self-regulation. Fifth, the study was based on a sample in Sichuan, which limits the generalizability of the findings to other parts of China.

Despite these limitations, our study was one of the first studies examining associations between socio-ecological factors and LTPA in older adults, and including self-regulation and social capital.

### Implications

We found that self-regulation mediated the pathway from social capital to physical activity. This finding may provide new ideas for the design of LTPA intervention programs for older adults. For example, considering the sharply reduced health effects of social capital in older adults over the age of 80 [15], interventions focused on self-regulation instead of social capital may be designed to improve physical activity and in turn to promote health in this population.

We also found that factors from three levels (individual, interpersonal, and social levels) were significantly associated with LTPA. A combination of individual level, interpersonal level, and social level factors should be considered in future physical activity interventions.

## Conclusions

In the study, we found that self-efficacy, perceived benefits and barriers, self-regulation, and social capital were significantly associated with LTPA while perceived physical environment was not significantly associated with LTPA in older adults. Self-regulation mediated the pathway from social capital to LTPA. These findings suggest that programs promoting LTPA in older populations based on socio-ecological model are most likely to be successful. The finding that self-regulation was not only a direct impact factor of LTPA but also a mediator between social capital and LTPA, may suggest that self-regulation should receive more attention in future interventions in this group. Longitudinal studies are needed to verify such findings.

## Supplementary Information

**Additional file 1.** Effects of individual level variables, self-regulation, social capital, and perceived physical environment on LTPA. There were no significant association of perceived physical environment with LTPA; *ρ < 0.05, ***ρ < 0.001.

**Additional file 2.** Effects of individual level variables, self-regulation and social capital on LTPA in older adults. The path from individual level to self-regulation was added in the model. *ρ < 0.05, ***ρ < 0.001.

## Data Availability

The datasets used and/or analysed during the current study are available from the corresponding author on reasonable request.
