# Supplementary material for: Association between socio-ecological factors and leisure time physical activity (LTPA) among older adults in Sichuan, China: a structural equation modeling analysis
Source: BMC Geriatr. 2022 Jan 18;22:60. doi: 10.1186/s12877-021-02730-9 (PMC8767736; doi:10.1186/s12877-021-02730-9)
Supplement: Supplementary file 1 — Additional file 1. Effects of individual level variables, self-regulation, social capital, and perceived physical environment on LTPA. There were no significant association of perceived physical environment with LTPA; *ρ < 0.05, ***ρ < 0.001. [file 12877_2021_2730_MOESM1_ESM.docx]

Figure S1. Effects of individual level variables, self-regulation, social capital, and perceived physical environment on LTPA.

Note: There were no significant association of perceived physical environment with LTPA; *ρ < 0.05, ***ρ < 0.001.
