# Supplementary material for: Association between socio-ecological factors and leisure time physical activity (LTPA) among older adults in Sichuan, China: a structural equation modeling analysis
Source: BMC Geriatr. 2022 Jan 18;22:60. doi: 10.1186/s12877-021-02730-9 (PMC8767736; doi:10.1186/s12877-021-02730-9)
Supplement: Supplementary file 2 — Additional file 2. Effects of individual level variables, self-regulation and social capital on LTPA in older adults. The path from individual level to self-regulation was added in the model. *ρ < 0.05, ***ρ < 0.001. [file 12877_2021_2730_MOESM2_ESM.docx]

Figure S2. Effects of individual level variables, self-regulation and social capital on LTPA in older adults.

Note: The path from individual level to self-regulation was added in the model.

*ρ < 0.05, ***ρ < 0.001.
